# Mother–infant interaction quality and sense of parenting competence at six months postpartum for first-time mothers in Taiwan: a multiple time series design

**DOI:** 10.1186/s12884-018-1979-7

**Published:** 2018-09-06

**Authors:** Fen-Fang Chung, Gwo-Hwa Wan, Su-Chen Kuo, Kuan-Chia Lin, Hsueh-Erh Liu

**Affiliations:** 1 Department of Nursing, College of Nursing, Chang Gung University of Science and Technology, Taoyuan, Taiwan; 2 Department of Nursing, Linkuo Chang Gung Memorial Hospital, Taoyuan, Taiwan; 3 Department of Respiratory Therapy, College of Medicine, Chang Gung University, 259, Wen-Hwa 1st Road, Kwei-Shan, Taoyuan, 333 Taiwan, Republic of China; 4 Department of Obstetrics and Gynecology, Taipei Chang Gung Memorial Hospital, Taipei, Taiwan; 5 Department of Respiratory Care, Chang Gung University of Science and Technology, Chiayi, Taiwan; 6 Department of Midwifery and Women Health Care, National Taipei University of Nursing and Health Sciences, Taipei, Taiwan; 7 Institute of Hospital and Health Care Administration, Community Research Center, National Yang-Ming University, Taipei, Taiwan; 8 School of Nursing, College of Medicine, Chang Gung University, 259, Wen-Hwa 1st Road, Kwei-Shan, Taoyuan, 333 Taiwan, Republic of China; 9 Department of Rheumatology, Linkuo Chang Gung Memorial Hospital, Taoyuan, Taiwan

**Keywords:** Parenting education, First-time mothers, Parent–infant interaction quality, Parenting sense of competence

## Abstract

**Background:**

For first-time mothers, not knowing how to interact with newborn infants increases anxiety and decreases the quality of the parent–infant interactions. A substantial lack of interactional knowledge can ultimately limit the adjustments necessary for a stable transition into motherhood. This study investigated how postpartum parenting education influenced first-time mothers’ mother–infant interaction quality and parenting sense of competence.

**Methods:**

Eighty-one healthy first-time-mother and infant dyads were recruited. The control group (*n* = 40) received postpartum care based on the medical and cultural norms practiced in Taiwan, while the experimental group (*n* = 41) received, on top of typical care, education by way of a 40-min videotape on infant states, behaviors, and communication cues, as well as a handout on play practices. Data were collected at five points: within the first week, and during follow-ups in the first, second, third, and sixth months after birth. We administered the Chinese versions of the Parenting Sense of Competence Scale and Edinburgh Perinatal Depression Scale, and used the Nursing Child Assessment Teaching Scale to score videotaped mother–infant interactions.

**Results:**

We observed an increase in the quality of mother–infant interaction within the experimental group only. Furthermore, at the five assessment points, we observed no significant changes in perceived parenting competence. Among all subjects, there were correlations between postpartum depression scores, parenting competency, and quality of mother–infant interaction.

**Conclusions:**

Our results indicate that first-time mothers in Taiwan who are provided with extra education on infants’ abilities and how to effectively play with infants are likely to exhibit improvements in quality of interaction.

**Electronic supplementary material:**

The online version of this article (10.1186/s12884-018-1979-7) contains supplementary material, which is available to authorized users.

## Background

The mother–infant interaction is the first and most important intrapersonal interaction that deeply affected a trust-building relationship in life [1]. It has substantial influences on infants’ language development, [2] emotional regulation, [3] and cognitive development [4, 5], while children whose mothers regularly engage in quality interaction with them tend to exhibit a high mental development index at 2 years old [6]. If the parents’ behavior shows strong intent for interaction, it attracts the attention of infants and initiates mutual exchange, response, and participation [7, 8]. In such cases, the infants actively contribute to social engagements and learn from these to anticipate social responses from caregivers [7]. Parent–child interaction is a shared, reciprocal experience within the dyads, whereby the experience of each has an impact on the experience of the other [7]. Effective parent–child interaction requires that both the infant and parent (or caregiver) send clear cues and respond to each other, thus facilitating the development of an interactive environment that continues the interaction [9, 10]. The parent and infant learn to adapt, modify, and change their behaviors in response to the other in every interaction process [11].

Researchers have determined that educational programs can be helpful means of improving first-time mothers’ abilities to interact with their infants [12–17]. Dickie and Gerber indicate that providing information on infant development, temperament, and cues in 4- to 12-month-old infants to their parents during a 16-h class was helpful in understanding the demands of newborns, providing appropriate contingent responses for parents, and producing feedback and increasing behavior predictability for infants [18]. A randomized controlled trial, based on Brazelton’s Neonatal Behavior Assessment Scale (NBAS) intervention, showed that a one-hour video and discussion on the behaviors and status of newborns for first-time mothers, delivered within 1 month postpartum, can increase the quality of mother–infant interactions. The results of this trial are based on the effect of information presented, about the newborn’s competence to interact, on a mother’s sensitive responsiveness toward her infant, thus promoting affectionate handling of the infant and motivating the mothers to become more involved and interact with their infants [15]. Similarly, another study similarly showed that a 45-min educational video on newborn behaviors for first-time pregnant women at 38 weeks’ gestation enhanced the quality of mother–infant interaction during the 24 h following birth. More specifically, the scores for newborns’ sensitivity to cues and socioemotional growth were found to be higher in the experimental group than in the control group that did not watch the video [12].

Furthermore, postpartum women must not only recover physically, but also, via mother–infant interaction, learn the skills necessary for caring for and identifying with their newborns, consequently developing their maternal role and behavior [19, 20]. The lack of understanding of newborns can increase mothers’ anxiety, thereby influencing mother–infant interaction [12, 21–23], maternal confidence [22, 24–26], and even mothers’ adaption to the maternal role [27]. Clark and Affonso report that it is exceedingly important for first-time mothers, in the first month after delivery, to deal with the gap in expectations and reality of maternal life, improve their own parenting skills, and establish a good relationship with the newborn [28].

Parenting sense of competence (PSOC) refers to the subjective feeling of a mothers’ ability to take care of their infants and understand their infants’ needs [29]. For first-time mothers, the most confusing period is 1–2 weeks after discharge from the hospital, after which their self-confidence in motherhood tends to increase [30–33]. Maternal confidence and ability tends to increase with time after birth, with the highest confidence and ability appearing at 4 months postpartum [34]. Improving mothers’ parenting skills can facilitate and enhance their sense of competence and satisfaction with the maternal role, and can prevent postnatal depression that fostering positive neonates’ psychosocial development [29]. However, PSOC is not related to mothers’ actual parenting abilities [35–37].

At 6 months postpartum, the postpartum depression symptoms can interfere with perceived parenting knowledge [38]. Women with postpartum depression showed a lower sense of mother role competence and satisfaction [29]. A Japanese study showed that mothers with higher postpartum depression score were more likely to engage in neglectful or aggressive parenting behaviors [39]. The depressed mothers were likely to show more negative and less positive behavior toward their infants than did the nondepressed mothers [40]. A negative correlation was found between the postpartum depression scores and PSOC [41–43] and parent–infant interaction quality [40, 44–46].

In Taiwan, women giving birth in the hospital are usually discharged three or 5 days after birth, depending on normal spontaneous delivery (NSD) or cesarean section (C/S). General postpartum care provided by hospitals concentrates on mothers’ physical care, observation of urine and stool, instructed uterine massage, normal change in lochia, perineal douche, postnatal exercises, episiotomy or C/S wound care, neonatal bathing, and treatment of physical problems. In Taiwan, few hospitals provided postpartum care, but even so, postpartum home visits focused on lactation consultation for promoting the breastfeeding behavior. It rarely provided parents the information about how to be sensitive to child’s signals, interpret them, and respond with emotional attunement. Previous studies have primarily focused on parenting stress and maternal confidence in Taiwanese postpartum women [30, 32]. Furthermore, Chinese typically express their emotions in more subtle forms than Western people. Chinese parents rarely praise their children verbally, because their culture encourages modesty and humility. Thus, human cultural factors influence beliefs and expressive behaviors in interaction [5, 47, 48]. Therefore, this study evaluated how mother-infant interactions and parenting competence are affected by a parenting competence education and how they change as the infant matures up to 6 months postpartum in Taiwan.

## Methods

### Study design and population

This study used a single-blind multiple time series design. In Taiwan, when visiting the doctor, pregnant women who come for prenatal examination and postpartum women who come for postnatal 6 weeks examination sit in the same waiting area. The pregnant and postpartum women could share and discuss the information of this study with each other. To avoid affecting the authenticity of the data, we approached the potential participants for the experimental group after the last postpartum woman in the control group had finished her postnatal 6 weeks examination. In other words, after the last participant in the control group had completed her postnatal examination, the experimental group recruitment began.

Study subjects were recruited from a 260-bed medical center in northern Taiwan between August 1, 2010 and February 2, 2012. The inclusion criteria were (1) primiparas; (2) 20–34 years old; (3) normal term and singleton birth; (4) no diagnosed complications of gestational diabetes mellitus, pregnancy-induced hypertension, preterm birth, placental abruption, postpartum hemorrhage, postpartum thrombophlebitis, or perinatal depression; (5) understood and spoke Mandarin or Taiwanese; (6) lived in northern Taiwan; and (7) signed an informed consent form. The necessary sample size was calculated by considering mother–infant interaction score as the primary outcome (Cohen’s d = 0.50) [8], while incorporating a 10% attrition rate. Given a 90% power and 5% type 1 error, the sample size was 82 dyads. This study received institutional review board (IRB) approval (no. 99-0243B).

### Materials

This study provided self-compiled CDs and manuals of “Tips on caring for your baby” to the postpartum women in the experimental group. The 40-min content of the CD was modified from the self-instructional video series entitled “Keys to caregiving” on infant, behaviors, communication cues, and state modulation [49]. Then, we translated the sub-titles of the film to Chinese. The content of the manuals mainly introduced the sensory development of newborns aged 0–6 months and summarized the CD’s content.

### Instruments

We conducted the study using two instruments: the Nursing Child Assessment Teaching Scale (NCATS) and the Chinese version of the Parenting Sense of Competence (C-PSOC) Scale. In addition, we collected data from Additional file 1 (Questionnaires A. demographic and background information) and Additional file 2 (Questionnaires B. breastfeeding and baby care practices), and at the same time used the Chinese version of the Edinburgh Perinatal Depression Scale (C-EPDS) to monitor postpartum depression.

The NCATS is a standardized tool for assessing interactions between caregivers and infants aged between 0 to 36 months [11, 16]. The NCATS was widely used in research and clinical practice for families and young children [2, 12, 17, 43, 50]. The NCATS consists of 73 binary items, divided into six subcategories: the caregiver’s sensitivity to cues, response to distress, social-emotional growth-fostering behaviors, and cognitive growth-fostering behaviors; the child’s clarity of cues and responsiveness to caregiver. In addition, the tool captures contingency items, such as the behavior of one member of the dyad affects the response from the other. The scale showed good internal consistency (0.76–0.87) and 4-week test-retest reliability (0.55–0.85) [11]. The Cronbach’s α of caregiver’s and child’s subscales in this study were 0.84 and 0.76, respectively.

The PSOC Scale was developed in 1978 [51] and the C-PSOC Scale. The latter had been validated with 0.85 internal consistency and 0.87 4-week test-retest reliability in a sample of Hong Kong Chinese mothers. Significant correlations with measures of self-esteem (*r* = 0.60, *p* <  0.01) and depression (*r* = − 0.48, *p* <  0.01) demonstrated good construct validity [52]. The Cronbach’s α of the PSOC Scale in this study was 0.90.

The questionnaires B were developed by the researcher for this study. The questionnaires B was collected from the postpartum mothers who self-reported that they were primary caregivers or not, and recalled how much time they spent to care for and handle with their babies in a week. The content validity was done by five obstetrics experts, who were asked to rate each item based on relevance, clarity, simplicity, and ambiguity on the 5-point Likert scale. The Content Validity Index (CVI) was 0.86–0.94.

The C-EPDS, which was developed by Cox, Holden, and Sagovsky in 1987 [53], has been validated in a sample of Taiwanese mothers [54]. The Cronbach’s α of C-EPDS in this study was 0.79. The 12/13 cutoff point was used to monitor postpartum depression [55] in our study. If necessary, the subjects were referred to the psychiatrist for further diagnosis, and if they were diagnosed with postpartum depression, the data collection was stopped and the subjects were excluded.

### Experimental procedure and data collection

One researcher identified women from the prenatal and delivery records who met the inclusion criteria and approached them in the postnatal ward at least 3 days after delivery (before they were discharged). Potential participants were first given written and verbal information about the study and then written informed consent was obtained. The mothers who agreed to participate in the study were asked to provide their addresses and phone numbers to contact them for their first visit after discharge. Their contact information was kept confidential for the purpose of the study.

The participants were visited five times – within the first week, the 1st month, the 2nd month, the 3rd month, and the 6th month after delivery – in postpartum nursing centers and/or their homes. Most Taiwanese conduct the ritual ‘Do the months’ (30-day period) after delivery [56], a form of support for postpartum women that originated in the Chinese culture [57]. Either the woman’s mother or mother-in-law assists her by taking care of her personal needs, helping her care for the newborn baby, taking care of any other children, and doing the housework [58]. Nowadays, around half of the new mothers choose to stay in postpartum nursing centers in northern Taiwan. It is a professional home-like health care facility operated by registered nurses. They provide postpartum services (for about a month) to facilitate the recovery of a postpartum woman and take care of her newborn baby, thus, conducting the ‘Doing the month’ ritual.

**Fig. 1:**
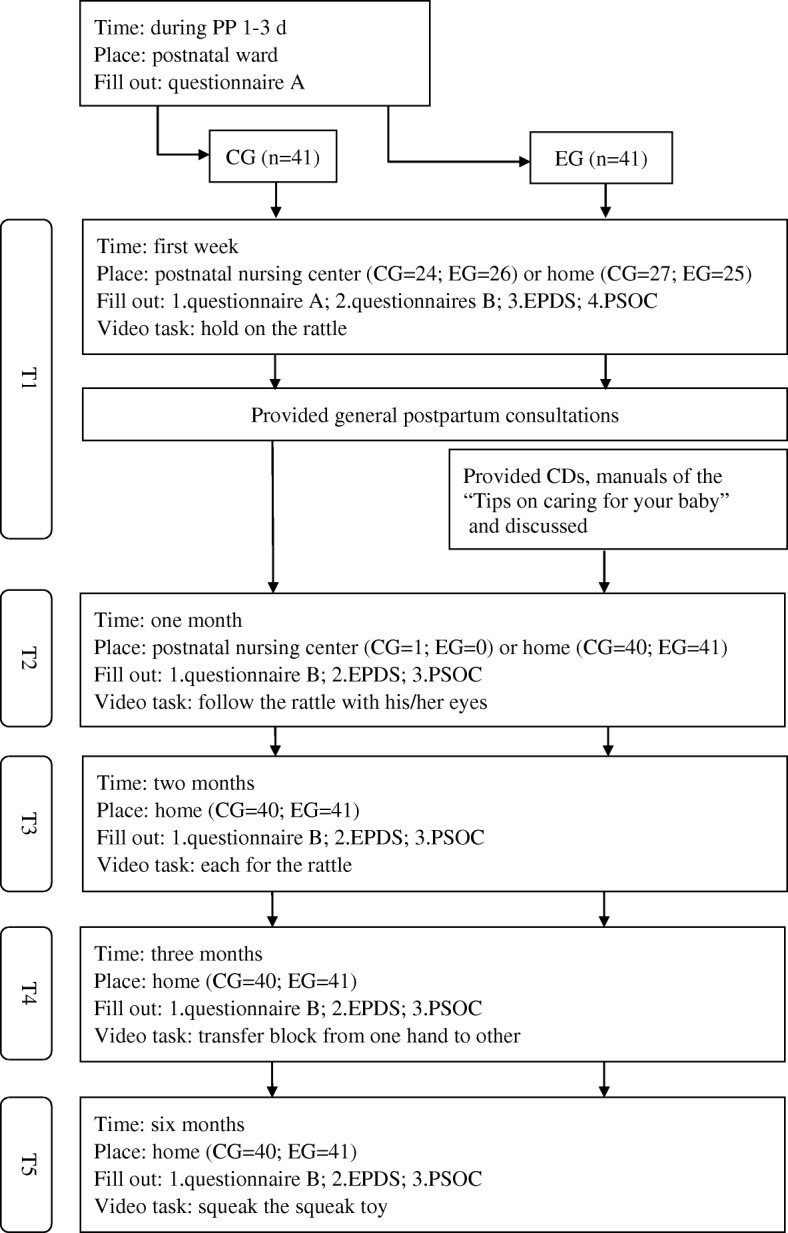
Experimental procedure and data collection

Each visit concluded with the administration of the above mentioned self-report questionnaires. The data collection procedure is presented in Fig. 1. When the baby was ready (in quiet alert state), the researcher (first author) introduced the task by giving a toy (rattle, block, or squeak toy) to the mother to play with her baby. We provided a new toy in each visit for hygiene considerations. To assess mother–infant interactions, we videotaped first-time mothers when they introduced new toys to their children and completed the task and when they needed to soothe their children. We videotaped for as long as the mothers interacted with their infants, which ranged from 2 to 16 min, depending on the actual interaction time until the mothers claimed they had done with teaching their infants [11]. The scoring was done by first researcher, certified in the use of NCATS tools (achieved an inter-rater reliability score of 90%). In this study, all toys, missions, and administration were taken and followed from the “Caregiving/Parent–Child Interaction Teaching Manual” as per NCATS protocol [11].

After videotaping, both the groups were given general postpartum guidance: (1) demo bathing of the baby and umbilical cord care skills, manually expressing breastmilk skills; (2) discussing breastfeeding problems and selection of breastfeeding pumps, to relieve baby gas/red spot on baby’s face; (3) providing the information on adding food to the baby’s diet; (4) listening and providing emotion support. However, there was no discussion of mother–infant interaction or parenting competence in the control group. The data collection procedure was conducted by a specific researcher to reduce the data collection bias and ensure the privacy and safety of videotaped information.

### Statistical analysis

All statistical analyses were conducted using SPSS Statistics 21.0 (IBM Corp., Armonk, NY, USA). The significance level for all tests was 0.05. The independent two-sample *t* test was used to identify the group differences in normally distributed continuous variables, while the chi-square test was used to determine group differences in categorical variables. The generalized estimating equation (GEE) analysis is a very useful technique when subject attrition threatens the power of longitudinal studies [59, 60]. In this study, we examined the trends in dependent variables, mother–infant interaction (NCATS) and PSOC using the GEE model, and set up “within-subject variable” = time; “covariance matrix” = robust estimator; “working correlation matrix” = exchangeable; “predictors factor” = group, time, and group time; “model” = group, time, group time and propensity scores. The propensity scores were calculated for parents’ age, education, and work status; mothers’ body mass index (BMI) before pregnancy, weight gain during pregnancy, and abortion history; whether the pregnancy was planned; mothers’ parenting knowledge; delivery method; and child’s gender and birth weight.

## Results

### Participants

Eighty-one primiparous mothers (40 in control group and 41 in experimental group) and their newborn infants were recruited for the study. One subject withdrew after 1 month because her mother-in-law held traditional views of parenting, such as newborns do not need much interaction or play. The dropout rate was 1.2%. No mother was referred to the psychiatrist in our study.

In the control group, around 30% of first-time mothers held no religious beliefs and only three mothers had ever had a smoking habit. In the experimental group, 17.3% held no religious beliefs, and two participants previously had smoking and drinking habits, while one still had a smoking habit (about 10 cigarettes per day). The BMI before pregnancy and the weight gain during pregnancy were in the normal range for both the groups. Approximately 93% of the subjects had obtained parenting information during their pregnancy, but none of this education had covered the topics taught in this study. Although infants’ body weight was significantly heavier in the control group than in the experimental group, the other basic characteristics and pregnancy/delivery data did not differ significantly (see Table 1).

**Table 1:** Personal characteristics and pregnancy/delivery information

	CG (*n* = 40)	EG (*n* = 41)	*t* / χ^2^ value	*p* value
Mother’s age, yrs	32.35 ± 2.80	33.49 ± 3.38	2.720	0.103
BMI, kg/m^2^	20.46 ± 2.17	20.49 ± 3.03	0.002	0.962
BW increase during pregnancy, kg	14.51 ± 3.90	15.29 ± 6.56	0.430	0.514
Father’s age, yrs	34.68 ± 3.94	36.15 ± 4.39	2.517	0.117
Infant BW, kg	3278.38 ± 377.06	3096.34 ± 371.54	4.789	0.032
Mother’s education			2.483	0.283
High school	1 (2.5)	4 (9.8)		
Junior college	26 (65.0)	21 (51.2)		
College or above	13 (32.5)	16 (39.0)		
Mother’s work status			2.614	0.282
Housewife	6 (15.0)	10 (24.4)		
Part-time Job	2 (5.0)	0		
Full-time job	32 (80.0)	31 (75.6)		
Father’s education			3.952	0.165
High school	0	4 (9.7)		
Junior college	20 (50.0)	17 (41.5)		
College or above	20 (50.0)	21 (48.8)		
Father’s work status			1.307	0.513
None	1 (2.5)	0		
Part-time Job	3 (7.5)	2 (4.9)		
Full-time job	36 (90.0)	39 (95.1)		
Abortion history	12 (30.0)	5 (12.2)	3.871	0.049
Pregnancy planned	27 (67.5)	29 (70.7)	0.099	0.753
Received parenting information	37 (92.5)	38 (92.7)	0.001	1.000
Delivery			2.102	0.147
NSD	16 (40.0)	23 (56.1)		
C/S	24 (60.0)	18 (43.9)		
Infant gender			0.307	0.579
Boy	21 (52.5)	19 (46.3)		
Girl	19 (47.5)	22 (53.7)		

*CG* control group, *EG* experimental group

### Infant care pattern in six months postpartum

Sixty percent of the control group and 65.9% of the experimental group chose a postpartum nursing center or a babysitter to assist in infant care during the first month postpartum. All first-time mothers self-reported that they spent a considerable amount of time on infant care during that first month. The exclusive breastfeeding rate was 58% in control group and 59% in experimental group. After 6 months, the infant care time and exclusive breastfeeding rates had reduced in both groups. There was no difference in infants’ time schedules, caregiving patterns, milk-feeding types, or mothers’ perceived resting frequency across any of the points in time or between groups (see Table 2).

**Table 2:** Infant care pattern during six months postpartum

	1st week	1st month	2nd month	3rd month	6th month
Baby average day wake time, hr./ day					
CG	5.3 ± 2.0	5.5 ± 2.7	5.6 ± 2.1	6.5 ± 2.4	8.8 ± 2.3
EG	4.8 ± 2.0	5.2 ± 2.6	5.9 ± 2.3	7.3 ± 2.1	9.0 ± 2.4
*p* value	0.281	0.636	0.581	0.124	0.758
Baby primary caregiver, *n* (%)					
CG					
Mother	18 (50.0)	37 (92.5)	26 (66.7)	13 (32.5)	10 (25.0)
Family	1 (2.8)	2 (5.0)	7 (17.9)	16 (40.0)	17 (42.5)
Others	17 (47.2)	1 (2.5)	6 (15.4)	11 (27.5)	13 (32.5)
EG					
Mother	13 (31.7)	40 (97.6)	31 (75.6)	19 (46.3)	16 (39.0)
Family	0	0	5 (12.2)	14 (34.1)	15 (36.6)
Others	28 (68.3)	1 (2.4)	5 (12.2)	8 (19.5)	10 (24.4)
*p* value	0.081	0.487	0.666	0.423	0.389
Care baby time per week, hr					
CG	119.6 ± 54.9	153.3 ± 38.7	138.7 ± 43.1	107.0 ± 47.1	100.4 ± 47.8
EG	123.8 ± 50.8	163.9 ± 20.6	148.4 ± 34.4	123.7 ± 47.2	121.1 ± 44.9
*p* value	0.730	0.127	0.271	0.115	0.048
Exclusive Breastfeeding, *n* (%)					
CG	19 (52.8)	23 (57.5)	17 (43.6)	18 (45.0)	19 (47.5)
EG	23 (56.1)	24 (58.5)	16 (39.0)	18 (43.9)	18 (43.9)
*p* value	0.770	0.925	0.678	0.921	0.745
Perceived resting frequency, *n* (%)					
CG					
Often	21 (58.3)	13 (32.5)	20 (51.3)	33 (82.5)	34 (85.0)
Sometimes	10 (27.8)	16 (40.0)	11 (28.2)	6 (15.0)	4 (10.0)
Seldom	5 (13.9)	11 (27.5)	8 (20.5)	1 (2.5)	2 (5.0)
EG					
Often	17 (41.5)	12 (29.3)	24 (58.5)	35 (85.4)	33 (80.5)
Sometimes	14 (34.1)	16 (39.0)	11 (26.8)	5 (12.2)	8 (19.5)
Seldom	10 (24.4)	13 (31.7)	6 (14.6)	1 (2.4)	0
*p* value	0.295	0.907	0.741	0.934	0.124

*CG* control group, *EG* experimental group

Most Chinese postpartum women undergo food therapy, that is, eating yang foods and avoiding yin foods, and engage in plenty of good rest in order to recover physically in the first month postpartum [58]. Common yang foods include chicken, egg, pig kidney, chicken or fish soup, ginger, and brown sugar. Common yin foods include most fresh fruits and vegetables, ice, and salt [61]. This diet is followed to compensate for the blood loss during childbirth [57].

### Trends in mother–infant interaction quality

**Fig. 2:**
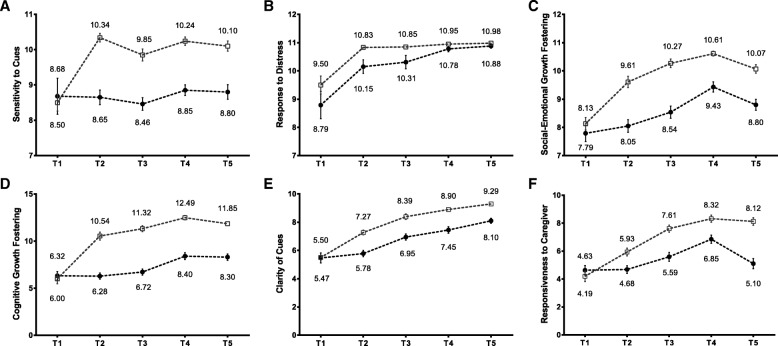
Trends for scores of the mother-infant interaction quality. T1: the 1st week; T2: the 1st month; T3: the 2nd month; T4: the 3rd month; T5: the 6th month after delivery; □: EG; ●: CG

**Fig. 3:**
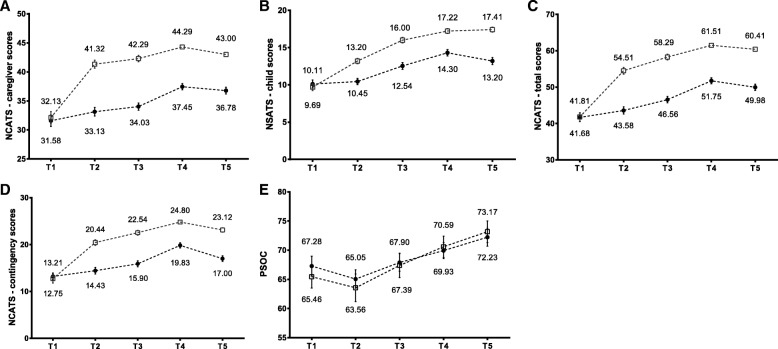
Effectiveness of postpartum parenting education on (**a**) mother, (**b**) infant, (**c**) total scores, (**d**) contingency scores, (**e**) PSOC. T1: the 1st week; T2: the 1st month; T3: the 2nd month; T4: the 3rd month; T5: the 6th month after delivery; □: EG; ●: CG

Figure 2 shows the results of the NCATS for caregivers and infants (caregivers: (sensitivity to cues, response to distress, social-emotional growth fostering, and cognitive growth fostering; infants: clarity of cues and responsiveness to caregiver). The sensitivity to cues score for caregivers showed a slight decreasing trend in the second (T3) and sixth (T5) months compared to the first week postpartum. Furthermore, the score of social-emotional growth fostering, cognitive growth fostering, and responsiveness to caregiver showed a decrease at 6 months. All the other scores for mother–infant interaction increased over time. Overall, the results indicated that the caregiver scores (Fig. 3a), child scores (Fig. 3b), total scores (Fig. 3c), and contingency scores (Fig. 3d) at the 1st to the 6th month were evidently higher in the experimental group than in the control group.

### Trends of the sense of parenting competence

We observed no difference in C-PSOC scores between the control group (mean = 67.28, SD = 10.18) and experimental group (mean = 65.46, SD = 12.56) in the first week (Fig. 3e). The C-PSOC scores then showed a decrease (control group: mean = 65.05, SD = 10.06; experimental group: mean = 63.56, SD = 15.15) at the 1st month, before gradually increasing from the 2nd to the 6th month. At no point were the C-PSOC scores significantly different between the two groups.

### Effectiveness on PCI and PSOC

This study evaluated the intervention effect on mother–infant interaction quality and PSOC using a GEE model (Table 3). We found no differences in sensitivity to cues, response to distress, social-emotional growth fostering, cognitive growth fostering, clarity of cues, or responsiveness to caregiver in the first week between the two groups. Notably, caregiver scores, child scores, total scores, and contingency scores for infants in the experimental group did not significantly differ from those in the control group. For the experimental group, a significant increasing trend was observed in sensitivity to cues, social-emotional growth fostering, cognitive growth fostering, clarity of cues, and responsiveness to caregiver, as well as overall caregiver scores, child scores, total scores, and contingency scores at the 1st month, the 2nd month, the 3rd month, and the 6th month. This indicated that receiving postpartum parenting education led to an increase in mother–infant interaction quality among first-time mothers in the 6 months after delivery, except for the aspect of interaction related to response to distress.

**Table 3:** Effectiveness of postpartum parenting education on the quality of mother-infant interaction and sense changes of parenting competence

	NCATS											
	Sensitivity to Cues				Response to Distress				Social-Emotional Growth Fostering			
	*B*	*S.E.*	Wald *χ*^*2*^	*p* value	*B*	*S.E.*	Wald *χ*^*2*^	*p* value	*B*	*S.E.*	Wald *χ*^*2*^	*p* value
Intercept	8.68	0.29	924.22^**^	< 0.001	8.78	0.48	328.50^**^	< 0.001	7.80	0.29	718.56^**^	< 0.001
EG^a^	−0.31	0.35	0.77	0.381	0.49	0.54	0.82	0.366	0.26	0.35	0.57	0.452
Time												
1st m vs. 1st wk^b^	−0.10	0.29	0.12	0.728	1.25	0.48	6.69^*^	0.010	0.18	0.30	0.38	0.539
2nd m vs. 1st wk^b^	−0.29	0.30	0.94	0.334	1.42	0.49	8.32^**^	0.004	0.66	0.31	4.60^*^	0.032
3rd m vs. 1st wk^b^	0.10	0.31	0.10	0.751	1.88	0.46	16.92^**^	< 0.001	1.56	0.31	25.09^**^	< 0.001
6th m vs. 1st wk^b^	0.05	0.28	0.03	0.866	1.98	0.46	18.83^**^	< 0.001	0.93	0.34	7.72^**^	0.005
Interaction												
EG × [1st m vs. 1st wk]^c^	1.93	0.36	28.74^**^	< 0.001	0.08	0.57	0.02	0.890	1.24	0.35	12.60^**^	< 0.001
EG × [2nd m vs. 1st wk]^c^	1.63	0.39	17.53^**^	< 0.001	−0.06	0.57	0.01	0.913	1.42	0.37	14.60^**^	< 0.001
EG × [3rd m vs. 1st wk]^c^	1.63	0.41	15.75^**^	< 0.001	−0.42	0.55	0.60	0.438	0.86	0.37	5.34^*^	0.021
EG × [6th m vs. 1st wk]^c^	1.53	0.37	16.85^**^	< 0.001	−0.50	0.55	0.83	0.364	0.95	0.41	5.48^*^	0.019
Propensity score	0.21	0.22	0.89	0.347	0.35	0.31	1.28	0.258	0.18	0.28	0.42	0.518
	NCATS											
	Cognitive Growth Fostering				Clarity of Cues				Responsiveness to Caregiver			
	*B*	*S.E.*	Wald *χ*^*2*^	*p* value	*B*	*S.E.*	Wald *χ*^*2*^	*p* value	*B*	*S.E.*	Wald *χ*^*2*^	*p* value
Intercept	6.67	0.45	222.11^**^	< 0.001	5.53	0.36	242.18^**^	< 0.001	4.77	0.36	179.79^**^	< 0.001
EG^a^	−0.23	0.67	0.15	0.736	0.03	0.38	0.01	0.941	−0.30	0.52	0.33	0.566
Time												
1st m vs. 1st wk^b^	−0.34	0.45	0.55	0.459	0.29	0.39	0.56	0.453	0.03	0.45	0.00	0.957
2nd m vs. 1st wk^b^	0.10	0.45	0.05	0.830	1.47	0.38	14.89^**^	< 0.001	0.95	0.45	4.53^*^	0.033
3rd m vs. 1st wk^b^	1.79	0.52	11.62^**^	0.001	1.97	0.43	21.15^**^	< 0.001	2.20	0.45	24.24^**^	< 0.001
6th m vs. 1st wk^b^	1.69	0.54	9.88^**^	0.002	2.62	0.40	42.71^**^	< 0.001	0.45	0.54	0.69	0.407
Interaction												
EG × [1st m vs. 1st wk]^c^	0.54	0.70	41.62^**^	< 0.001	1.51	0.45	11.38^**^	0.001	1.67	0.62	7.32^**^	0.007
EG × [2nd m vs. 1st wk]^c^	4.89	0.69	49.60^**^	< 0.001	1.45	0.46	9.77^**^	0.002	2.42	0.61	15.70^**^	< 0.001
EG × [3rd m vs. 1st wk]^c^	4.37	0.75	34.30^**^	< 0.001	1.47	0.47	9.95^**^	0.002	1.88	0.63	9.05^**^	0.003
EG × [6th m vs. 1st wk]^c^	3.83	0.72	27.96^**^	< 0.001	1.21	0.44	7.37^**^	0.007	3.44	0.74	21.75^**^	< 0.001
Propensity score	−0.17	0.54	0.10	0.754	−0.13	0.24	0.29	0.590	3.35	0.43	0.67	0.413
	NCATS											
	Caregiver scores				Child scores				Total scores			
	*B*	*S.E.*	Wald *χ*^*2*^	*p* value	*B*	*S.E.*	Wald *χ*^*2*^	*p* value	*B*	*S.E.*	Wald *χ*^*2*^	*p* value
Intercept	32.01	0.97	1095.92^**^	< 0.001	10.30	0.56	343.17^**^	< 0.001	42.30	1.16	1328.29^**^	< 0.001
EG^a^	0.19	1.30	0.02	0.887	−0.27	0.71	0.15	0.702	−0.11	1.64	0.01	0.946
Time												
1st m vs. 1st wk^b^	0.92	0.92	1.00	0.318	0.31	0.69	0.20	0.652	1.25	1.29	0.93	0.336
2nd m vs. 1st wk^b^	1.81	0.97	3.49	0.062	2.42	0.64	14.45^**^	< 0.001	4.26	1.31	10.60^**^	0.001
3rd m vs. 1st wk^b^	5.24	1.03	26.03^**^	< 0.001	4.16	0.67	38.32^**^	< 0.001	9.42	1.41	44.64^**^	< 0.001
6th m vs. 1st wk^b^	4.57	1.00	21.08^**^	< 0.001	3.06	0.72	18.19^**^	< 0.001	7.65	1.32	33.57^**^	< 0.001
Interaction												
EG × [1st m vs. 1st wk]^c^	7.82	1.29	36.77^**^	< 0.001	3.18	0.90	12.43^**^	< 0.001	11.02	1.77	38.75^**^	< 0.001
EG × [2nd m vs. 1st wk]^c^	7.91	1.34	35.07^**^	< 0.001	3.87	0.87	19.98^**^	< 0.001	11.79	1.79	43.27^**^	< 0.001
EG × [3rd m vs. 1st wk]^c^	6.47	1.44	20.33^**^	< 0.001	3.35	0.87	14.99^**^	< 0.001	9.85	1.87	27.73^**^	< 0.001
EG × [6th m vs. 1st wk]^c^	5.85	1.39	17.68^**^	< 0.001	4.65	0.95	23.68^**^	< 0.001	10.52	1.82	33.57^**^	< 0.001
Propensity score	0.57	0.90	0.40	0.528	−0.49	0.55	0.78	0.376	0.84	1.14	0.01	0.941
	NCATS				PSOC							
	Contingency scores											
	*B*	*S.E.*	Wald *χ*^*2*^	*p* value	*B*	*S.E.*	Wald *χ*^*2*^	*p* value				
Intercept	13.67	0.75	336.23^**^	< 0.001	66.78	2.30	843.52^**^	< 0.001				
EG^a^	−0.36	1.15	0.10	0.751	−2.59	2.94	0.78	0.379				
Time												
1st m vs. 1st wk^b^	0.91	0.92	0.98	0.323	−2.37	1.27	3.47	0.062				
2nd m vs. 1st wk^b^	2.40	0.90	7.18^**^	0.007	0.15	1.13	0.02	0.896				
3rd m vs. 1st wk^b^	6.31	0.87	52.00^**^	< 0.001	2.50	1.24	4.09^*^	0.043				
6th m vs. 1st wk^b^	3.48	0.78	20.15^**^	< 0.001	4.80	1.41	11.56^**^	0.001				
Interaction												
EG × [1st m vs. 1st wk]^c^	6.53	1.26	26.85^**^	< 0.001	0.47	1.82	0.07	0.796				
EG × [2nd m vs. 1st wk]^c^	7.14	1.26	32.05^**^	< 0.001	1.78	1.74	1.05	0.306				
EG × [3rd m vs. 1st wk]^c^	5.50	1.31	17.62^**^	< 0.001	2.62	1.83	2.06	0.151				
EG × [6th m vs. 1st wk]^c^	6.64	1.29	26.51^**^	< 0.001	2.91	2.07	1.97	0.161				
Propensity score	−0.47	0.82	0.32	0.572	1.92	4.20	0.21	0.647				

^*^: *p* < 0.05, ^**^: *p* < 0.01; df = 1; ^a^ Reference category: CG; ^b^ Reference category: 1st week (Baseline); ^c^ Reference category: CG × 1st week (Baseline)

*NCATS* nursing child assessment teaching scale, *PSOC* parenting sense of competence, *EG* experimental group, *1st wk* the 1st week,*1st m* the 1st month; *2nd m* the 2nd month, *3rd m* the 3rd month, *6th m* the 6th month

The PSOC at baseline in the experimental group was similar to that in the control group. Furthermore, we observed no change in the PSOC in the experimental group between the 1st month and the 6th month, indicating that while the postpartum parenting education appeared to help improve mother–infant interaction quality, it has no effect on the PSOC of first-time mothers. Among all subjects, clear correlations were found between postpartum depression, parenting competency, and quality of mother–infant interaction (see Table 4).

**Table 4:** Associations between scores of postpartum depression, parenting competency, and quality of mother-infant interaction

Scores		PSOC	NCATS
EPDS	*r*_*s*_	−0.618^**^	−0.146^**^
	*p* value	< 0.001	0.006
PSOC	*r*_*s*_	1	0.156^**^
	*p* value	–	0.003

*PSOC* parenting sense of competence, *NCATS* nursing child assessment teaching scale, *EPDS* Edinburgh perinatal depression scale

^**^: *p* < 0.01

## Discussion

In this study, we evaluated how postpartum parenting education influenced the quality of mother–infant interaction and PSOC in the 6 months after delivery among first-time mothers in Taiwan. The education was evidently helpful in improving overall mother–infant interaction quality during the 6 months after delivery, but it had no effect on the response to distress aspect of that interaction. This result was in accordance with the findings of previous studies indicating that postpartum parenting education is effective in improving mother–infant interaction quality [12–15, 18, 62, 63]. It should be noted that, while the interaction scores of the mother’s response to infant’s distress in the first and second month after delivery were higher in the experimental group than in the control group, there was no significant difference in the magnitude of the progress between the two groups. A possible reason for this is that first-time mothers must be able to manage infants’ distress, for example, crying, being fussy, or generally upset in everyday life. As such, while the intervention education made the mothers in the experimental group learn these skills sooner, the control group mothers were still accumulating parenting experience during the first month after delivery, including how to respond to infants’ distress; thus, their abilities would not differ substantially from that of the experimental group. Previous studies have similarly found that first-time mothers apparently show improvements in knowledge of caring for newborns in the two–six weeks after birth [31–33].

However, when observing the influence of time on mother–infant interaction quality, response to distress showed apparent progress at the 1st month, the 2nd month, the 3rd month, and the 6th month, but there was no significant progress in sensitivity to cues over the assessment period. This suggests that mother–infant interaction quality exhibits positive changes through the accumulation of experience between mothers and infants. More specifically, response to distress showed a rather rapid increase, whereas sensitivity to cues might need greater contact time or special instructions. Recognizing that a child is a person in the environment is considered the basis of personal interaction [9]. Sumner and Spietz report that numerous first-time parents are unable to recognize their newborns’ abilities or interact with their infants as people in their environment [11]. First-time parents become familiar with the characteristics and abilities of their newborns through the process of caregiving [19, 20], which makes them more sensitive to newborns’ behaviors and demands and engage in more appropriate responses, thus improving interaction quality [10].

Gibaud-Wallston and Wandersman showed that the parenting competence of first-time parents began to increase from about 6 weeks after delivery [51]. Our results showed that postpartum education focusing on infants’ states, behaviors, and communication cues using videos and instruction manuals cannot improve PSOC during in the 6 months after delivery. We found that the lowest PSOC scores in both groups were in the first month postpartum, after which they increased until the third month. This is perhaps because, in the first month, first-time mothers spend considerable amount of time taking care of their children rather than recovering. During the first month home visit, we found that the mothers consistently asked questions about infants’ crying during the nighttime, bowel movements, and breastfeeding, as well as how to adjust infants’ time schedules. Thus, even if first-time mothers might have been thoroughly immersed in the joyful feeling of motherhood, they might have been experiencing feelings of anxiety, frustration, lack of competence, and exhaustion, thus leading to the low PSOC [64].

Past research has shown that newborns’ mothers were most stressed in the first month postpartum, after which their stress began decreasing as their understanding of infants, mastery of care skills, and perceived ability to care for the infant increased [34]. This result is similar to that of our own study, wherein PSOC in both the experimental and control groups increased over time, beginning from the second month of delivery. Additionally, mothers’ knowledge of newborn care and maternal confidence increased over time. This somewhat consistent with the findings of Wu (2000), who found that providing physical care for newborns, establishing a mother–infant relationship, and instructing first-time mothers on newborns’ conditions and behavioral cues during their discharge from the hospital could improve maternal confidence in the second week of postpartum; however, no difference in maternal confidence was found after the first month [31]. Other studies have also demonstrated similar results: Kuo et al. (2000) show that providing instructions on newborns’ physical care and behavioral status at postpartum can increase maternal confidence in the second week of postpartum, [32] while Chen (2005) shows that providing instructions on newborns’ development and physical care at 32–34 weeks of pregnancy could enhance maternal confidence at 6 weeks’ postpartum [33].

Additionally, there was no significant difference between the groups in the magnitude of the change in PSOC between the 1st month and 6th month. Previous studies have emphasized instruction on practical behaviors, such as skills in caring for newborns, as they can facilitate mothers’ self-confidence in their parenting skills and ability to understand infants’ demands. A possible reason for no significant difference in the magnitude of change was the C-PSOC, which evaluates both self-efficacy and self-satisfaction of mothers’ parenting role abilities and is associated more with mothers’ personal feelings, but is not representative of their actual abilities [36, 37]. Influenced by modesty and humility in Taiwanese culture [5], mothers underestimate themselves and the maternal role achievement needs more time than maternal skills [19, 20]. Additionally, higher depression scores were correlated with lower parenting competence scores and interaction scores. Also, higher interaction scores were correlated with higher parenting competence scores in this study. These results were similar to the previous studies [38, 39].

In this study, we could not randomly assign the participants to the two groups, and approximately 60% of the participants remained in postpartum nursing facilities for the first month. Thus, we employed the propensity score and GEE model for reducing the selection bias and the influence of missing data*.* Additionally, further study warrants investigations on the impact of mother–infant interaction quality with the total time of using the CDs/manuals and different child sequence.

We also found that first-time mothers usually focused on completing the task rather than giving new toys to their children for exploration, and seldom praised their children. It was found that 93% of the participants obtained information on raising children from relatives, friends, books, and magazines, other mothers, health care personnel, and the internet. Thus, it would seem necessary to educate Taiwanese first-time mothers on how to play with their infants, as well as how to be sensitive to infants’ behaviors. We suggest providing information about newborns’ characteristics, their ability to play with parents and grandparents, and postpartum nursing facilities via the internet as well as through service education.

## Conclusions

It was helpful in mother–infant interaction quality when the first-time mothers received the parenting educations on infants’ abilities and how to play with infants during the 6 months after delivery. An apparent progress on response to distress in mother–infant interaction quality was shown at the 1st, 2nd, 3rd, and 6th months, respectively. The lowest PSOC scores in both groups were found in the first month postpartum, after which they increased until the third month. Significant associations found among postpartum depression, parenting competency, and quality of mother–infant interaction in the two groups.

## Additional files

Additional file 1: Questionnaires A. demographic and background information. (PDF 347 kb)

Additional file 2: Questionnaires B. breastfeeding and baby care practices. (PDF 280 kb)

## Abbreviations

BMI: Body mass index
C-EPDS: Chinese version of the Edinburgh Perinatal Depression Scale
C-PSOC: Chinese version of the Parenting Sense of Competence Scale
GEE: Generalized estimating equation
NCATS: Nursing child assessment teaching scale
PSOC: Parenting sense of competence
SD: Standard deviation

## References

[1] Hofer MA (2006). Psychobiological roots of early attachment. Curr Dir Psychol Sci.

[2] Magill-Evans J, Harrison MJ (1999). Parent-child interactions and development of toddlers born preterm. West J Nurs Res.

[3] MacLean PC, Rynes KN, Aragón C, Caprihan A, Phillips JP, Lowe JR (2014). Mother-infant mutual eye gaze supports emotion regulation in infancy during the still-face paradigm. Infant Behav Dev..

[4] Shannon JD, Tamis-LeMonda CS, London K, Cabrera N (2002). Beyond rough and tumble: low-income fathers’ interactions and childrens’ cognitive development at 24 months. Parenting.

[5] Negayama K, Delafield-Butt JT, Momose K, Ishijima K, Kawahara N, Lux EJ, Murphy A, Kaliarntas K (2015). Embodied intersubjective engagement in mother–infant tactile communication: a cross-cultural study of Japanese and Scottish mother–infant behaviors during infant pick-up. Front Psychol.

[6] Barnard KE, Eyres SJ (1979). Child health assessment, part 2: the first year of life.

[7] Trevarthen C, Aitken K (2001). Infant Intersubjectivity: research, theory, and clinical applications. J Child Psychol Psychiatry.

[8] Brazelton TB, Koslowski B, Main M, Lewis M, Rosenblum LA (1974). The origins of reciprocity: the early mother-infant interaction. The effect of the infant on its caregiver.

[9] White C, Simon M, Bryan A (2002). Using evidence to educate birthing center nursing staff about infant states, cues, and behaviors. MCN Am J Matern Child Nurs.

[10] Barnard KE (1978). NCAST: nursing child assessment satellite training: learning resource manual.

[11] Sumner G, Spietz A (1994). NCAST caregiver/ parent-child interaction teaching manual.

[12] Leitch DB (1999). Mother-infant interaction: achieving synchrony. Nurs Res.

[13] Hall LA (1980). Effect of teaching on primiparas’ perceptions of their newborn. Nurs Res.

[14] Waterston T, Welsh B, Keane B, Cook M, Hammal D, Parker L, McConachie H (2009). Improving early relationships: a randomized, controlled trial of an age-paced parenting newsletter. Pediatrics.

[15] Wendland-Carro J, Piccinini CA, Millar WS (1999). The role of an early intervention on enhancing the quality of mother-infant interaction. Child Dev.

[16] Letourneau N (1997). Fostering resiliency in infants and young children through parent-infant interaction. Infants Young Children.

[17] Letourneau N, Drummond J, Fleming D, Kysela G, McDonald L, Stewart M (2001). Supporting parents: can intervention improve parent-child relationships?. J Fam Nurs.

[18] Dickie JR, Gerber SC (1980). Training in social competence: the effect on mothers, fathers, and infants. Child Dev.

[19] Rubin R (1984). Maternal identity and the maternal experience.

[20] Mercer RT (2006). Nursing support of the process of becoming a mother. J Obstet Gynecol Neonatal Nurs.

[21] Parfitt Y, Pike A, Ayers S (2013). The impact of parents’ mental health on parent-baby interaction: a prospective study. Infant Behav Dev.

[22] Sumner B, Barnard K (1980). Keys to caregiving.

[23] Nicol-Harper R, Harvey AG, Stein A (2007). Interactions between mothers and infants: impact of maternal anxiety. Infant Behav Dev..

[24] Pridham KF, Chang AS. Transition to being the mother of a new infant in the first 3 months: maternal problem solving and self-appraisals. J Adv Nurs. 1992;17(2):204–16. 10.1111/j.1365-2648.1992.tb01875.x 1556329

[25] Ngai FW, Chan SWC (2011). Psychosocial factors and maternal wellbeing: an exploratory path analysis. Int J Nurs Stud.

[26] Sayil M, Güre A, Uçanok Z (2006). First time mothers’ anxiety and depressive symptoms across the transition to motherhood: associations with maternal and environmental characteristics. Women Health.

[27] Guedeney A, Guedeney N, Wendland J, Burtchen N (2014). Treatment-mother-infant relationship psychotherapy. Best Pract Res Clin Obstet Gynaecol.

[28] Clark AL, Affonso DD (1979). Childbearing: A nursing perspective.

[29] Ngai FW, Chan SWC, Ip WY (2010). Predictors and correlates of maternal role competence and satisfaction. Nurs Res.

[30] Hong CH (2001). Postpartum stress and social support of women at different places of their confinement and at different points of time during the puerperium. Public Health Q.

[31] Wu HW (2000). The effects of discharge nursing instruction on primiparas’ knowledge and confidence in caring for newborn at home.

[32] Kuo SF, Chen YC, Mao HC, Tsou KI (2000). Effects of individual nursing instruction on infant care knowledge and maternal confidence of primiparas. J Nurs Res..

[33] Chen YS (2005). The effect of a prenatal web-based newborn care education on mother's newborn care knowledge, and maternal confidence.

[34] Liu CC, Chen YC, Yeh YP, Hsieh YS. Effects of maternal confidence and competence on maternal parenting stress in newborn care. J Adv Nurs. 2012;68(4):908–18. 10.1111/j.1365-2648.2011.05796.x. (in Chinese). 10.1111/j.1365-2648.2011.05796.x 21790741

[35] Gao LL, Xie W, Yang X, Chan SWC (2015). Effects of an interpersonal-psychotherapy-oriented postnatal programme for Chinese first-time mothers: a randomized controlled trial. Int J Nurs Stud.

[36] Walker LO, Crain H, Thompson E (1986). Mothering behavior and maternal role attainment during the postpartum period. Nurs Res.

[37] Zahr LK (1991). The relationship between maternal confidence and mother-infant behavior in premature infants. Res Nurs Health.

[38] Mohammad KI, Gamble J, Creedy DK. Prevalence and factors associated with the development of antenatal and postnatal depression among Jordanian women. Midwifery. 2011; 10.1016/j.midw.2010.10.008. 10.1016/j.midw.2010.10.008 21130548

[39] Sagami A, Kayama M, Senoo E (2004). The relationship between postpartum depression and abusive parenting behavior of Japanese mothers: a survey of mothers with a child less than one year old. Bull Menn Clin.

[40] Field T, Healy B, Goldstein S, Guthertz M (1990). Behaviour-state matching and synchrony in mother-infant interactions of nondepressed versus depressed dyads. Dev Psychol.

[41] Ngai FW, Chan SWC, Ip WY (2009). The effects of a childbirth psychoeducation program on learned resourcefulness, maternal role competence and perinatal depression: a quasi-experiment. Int J Nurs Stud.

[42] Gao LL, Chan SWC, Sun K (2012). Effects of an interpersonal-psychotherapy- oriented childbirth education programme for Chinese first-time childbearing women at 3-month follow up: randomised controlled trial. Int J Nurs Stud.

[43] Chung CC, Liu HE (2015). Mother-infant interaction quality and sense of parenting competence during two months postpartum for first time mothers. J Midwifery.

[44] Leadbeater BJ, Bishop SJ, Raver CC (1996). Quality of mother-toddler interaction, maternal depressive symptoms, and behaviour problems in preschoolers of adolescent mothers. Dev Psychol.

[45] Steadman J, Pawlby S, Mayers A, Bucks RS, Gregoire A, Miele-Norton M, Hogan AM (2007). An exploratory study of the relationship between mother-infant interaction and maternal cognitive function in mothers with mental illness. J Reprod Infant Psychol.

[46] Tronick E, Reck C (2009). Infants of depressed mothers. Harv Rev Psychiatry.

[47] Chao RK (1994). Beyond parental control and authoritarian parenting style: understanding Chinese parenting through the cultural notion of training. Child Dev.

[48] Liu M, Guo F (2010). Parenting practices and their relevance to child behaviors in Canada and China. Scand J Psychol.

[49] NCAST-AVENUW (2008). Keys to caregiving. revised ed.

[50] Bryan AA (2000). Enhancing parent-child interaction with a prenatal couple intervention. MCN Am J Matern Child Nurs.

[51] Gibaud-Wallston J, Wandersman LP (1978). Development and utility of the Parenting Sense of Competence Scale.

[52] Ngai FW, Chan SWC, Holroyd E (2007). Translation and validation of a Chinese version of the parenting sense of competence scale in Chinese mothers. Nurs Res.

[53] Cox JL, Holden JM, Sagovsky R (1987). Detection of postnatal depression. Development of the 10-item Edinburgh postnatal depression scale. Br J Psychiatry.

[54] Heh SS (2001). Validation of the Chinese version of the Edinburgh postnatal depression scale: detecting postnatal depression in Taiwanese women. J Nurs Res.

[55] Teng HW, Hsu CS, Shih SM, Lu ML, Pan JJ, Shen WW (2005). Screening postpartum depression with the Taiwanese version of the Edinburgh postnatal depression scale. Compr Psychiatry.

[56] Liu YQ, Petrini M, Maloni JA (2015). “doing the month”: postpartum practices in Chinese women. Nurs Health Sci.

[57] Heh SS (2004). “Doing the month” and social support. Fu-Jen. J Med.

[58] Hung CH, Yu CY, Ou CC, Liang WW (2009). Taiwanese maternal health in the postpartum nursing Centre. J Clini Nurs.

[59] Liang KY, Zeger SL (1986). Longitudinal data analysis using generalized linear models. Biometrika.

[60] Zeger SL, Liang KY (1986). Longitudinal data analysis for discrete and continuous outcomes. Biometrics.

[61] Ma GN, Bai GJ (2010). Dietary ying yang balance. Clin J Chin Med.

[62] Liptak GS, Keller BB, Feldman AW, Chamberlin RW (1983). Enhancing infant development and parent-practitioner interaction with the Brazelton neonatal assessment scale. Pediatrics.

[63] Myers BJ (1982). Early intervention using Brazelton training with middle-class mothers and fathers of newborns. Child Dev.

[64] Chen MI (1999). A primipara's experience as a new motherhood during the early postpartum period.

