# Supplementary material for: Mother–infant interaction quality and sense of parenting competence at six months postpartum for first-time mothers in Taiwan: a multiple time series design
Source: BMC Pregnancy Childbirth. 2018 Sep 6;18:365. doi: 10.1186/s12884-018-1979-7 (PMC6127995; doi:10.1186/s12884-018-1979-7)
Supplement: Supplementary file 1 — Questionnaires A. demographic and background information. (PDF 347 kb) [file 12884_2018_1979_MOESM1_ESM.pdf]

Questionnaire A

1. Name:__________________

   Phone numbers:__________________

   Address of first time visited:____

2. Mother’s age (m/y):_______

   Education: □(1) Junior high school or below □(2) Senior high school

   □(3) College or above

   Religion： □(1) None □(2) Buddhism □(3) I-Kuan Tao □(4) Taoism

   □(5) Islam □(6) Christian □(7) Catholicism □(8)Other:_____

   Work status: □(1) Housewife □(2) Part-time Job □(3) Full-time job

   Occupation: ______________

3. Father’s age (m/y): _________

   Education: □(1) Junior high school or below □(2) Senior high school

   □(3) College or above

   Religion： □(1) None □(2) Buddhism □(3) I-Kuan Tao □(4) Taoism

   □(5) Islam □(6) Christian □(7) Catholicism □(8)Other:_____

   Work status: □(1) Unemployment □(2) Part-time Job □(3) Full-time job

   Occupation: ______________

4. Pregnancy planned? □(1) None □(2) Yes

5. Obtained information on raising children during pregnancy?

□(1) None

□(2) Yes, from? □(a) Relatives or friends □(b) Books and magazines

□(c) Health care personnel □(d) Internet □(e) Other:______

6. Smoking habit? □(1) None □(2) Yes, ______cigarettes per day for ______years

□(3) Quit

7. Drinking habit? □(1) None □(2) Yes, ______glasses (240 c.c.) per day for____years

□(3) Quit

(*Item 8-9, recorded by a researcher)

8. Gravida_____ Term_____ Pre-term_____ Abortion______ Living______

BW (before pregnancy): _______ Kg; BW (before labor): _______ Kg;

Weight gain: _______ Kg

GA______wk

Delivery: □(1)NSD；□(2)C/S

Baby feeding: □(1)Breastfeeding □(2) Bottle-feeding

9. Newborn Birth Time: _____y_____m_____d,_____:_____

Gender: □(1)Boy □(2)Girl

Birth weight: _____________ gm; Apgar Score:______→______
