# Supplementary material for: Mother–infant interaction quality and sense of parenting competence at six months postpartum for first-time mothers in Taiwan: a multiple time series design
Source: BMC Pregnancy Childbirth. 2018 Sep 6;18:365. doi: 10.1186/s12884-018-1979-7 (PMC6127995; doi:10.1186/s12884-018-1979-7)
Supplement: Supplementary file 2 — Questionnaires B. breastfeeding and baby care practices. (PDF 280 kb) [file 12884_2018_1979_MOESM2_ESM.pdf]

Questionnaire B

1. Visited Place: □(1) Postpartum nursing centers □(2) Home

2. Baby average wake time per day: __________ hr/day

3. Baby primary caregiver: □(1) Mother □(2) Father □(3) Family □(4) Others

4. Mother care baby pattern: □(1) Day and night, 24hrs. □(2) After work

□(3) Weekend Only □(4) Others:___________

5. Mother spent to care for and handle with their babies in a week?__________hr/wk

6. Baby feeding: □(1) Exclusive Breastfeeding □(2) Exclusive Bottle-feeding

□(3) Mixed □(4) start eating Non-staple food

□(5) Others:_____

7. Mother perceived resting frequency: □(1) Often □(2) Sometimes □(3) Seldom
